# Impact of Hormone-Associated Resistance to Activated Protein C on the Thrombotic Potential of Oral Contraceptives: A Prospective Observational Study

**DOI:** 10.1371/journal.pone.0105007

**Published:** 2014-08-14

**Authors:** Heiko Rühl, Lars Schröder, Jens Müller, Shorena Sukhitashvili, Julia Welz, Walther C. Kuhn, Johannes Oldenburg, Christian Rudlowski, Bernd Pötzsch

**Affiliations:** 1 Institute of Experimental Hematology and Transfusion Medicine, University Hospital Bonn, Bonn, Germany; 2 Department of Gynecology and Obstetrics, Center for Integrated Oncology (CIO) Köln/Bonn, University Hospital Bonn, Bonn, Germany; University Hospital Medical Centre, Germany

## Abstract

**Introduction:**

The increased thrombotic risk of oral contraceptives (OC) has been attributed to various alterations of the hemostatic system, including acquired resistance to activated protein C (APC). To evaluate to what extent OC-associated APC resistance induces a prothrombotic state we monitored plasma levels of thrombin and molecular markers specific for thrombin formation in women starting OC use. Elevated plasma levels of thrombin have been reported to characterize situations of high thrombotic risk such as trauma-induced hypercoagulability, but have not yet been studied during OC use.

**Patients and Methods:**

Blood samples were collected prospectively from healthy women (n = 21) before and during three menstruation cycles after start of OC. APC resistance was evaluated using a thrombin generation-based assay. Plasma levels of thrombin and APC were directly measured using highly sensitive oligonucleotide-based enzyme capture assay (OECA) technology. Thrombin generation markers and other hemostasis parameters were measured additionally.

**Results:**

All women developed APC resistance as indicated by an increased APC sensitivity ratio compared with baseline after start of OC (p = 0.0003). Simultaneously, plasma levels of thrombin, prothrombin fragment 1+2, and of thrombin-antithrombin complexes did not change, ruling out increased thrombin formation. APC plasma levels were also not influenced by OC use, giving further evidence that increased thrombin formation did not occur.

**Conclusions:**

In the majority of OC users no enhanced thrombin formation occurs despite the development of APC resistance. It cannot be ruled out, however, that thrombin formation might occur to a greater extent in the presence of additional risk factors. If this were the case, endogenous thrombin levels might be a potential biomarker candidate to identify women at high thrombotic risk during OC treatment. Large-scale studies are required to assess the value of plasma levels of thrombin as predictors of OC-associated thrombotic risk.

## Introduction

Venous thrombosis (VT) is a major complication of combined oral contraceptive (OC) use [1]. Various epidemiological studies demonstrate a three- to seven-fold increased VT risk depending on the type of OC [2]. Careful assessment of the thrombotic risk is therefore recommended prior to prescription to reduce the rate of OC-related VT. In daily clinical practice, however, this is often challenging since appropriate laboratory assays predicting an increased thrombotic risk have been not available so far. There is therefore an urgent need to identify biomarkers that can be used to assess the OC-related VT risk. The development of such biomarkers requires a comprehensive knowledge on molecular mechanisms involved in the development of a hormone-induced prothrombotic phenotype.

The increased VT risk of OC use has been attributed to various alterations of hemostatis including dysfunctions of the protein C-(PC)-pathway. The PC pathway is initiated on the endothelial cell surface by complex formation of thrombomodulin and thrombin, the key enzyme of the coagulation cascade. In this complex thrombin converts PC into the active enzyme activated PC (APC). APC down-regulates further thrombin formation by proteolytic cleavage of the activated cofactors V (FV) and VIII (FVIII) [3], [4]. The clinical importance of this anticoagulant pathway is demonstrated by the increased thrombotic risk of patients with inherited deficiencies of PC, PS, or the FV-Leiden mutation [5]. This mutation abolishes an APC cleavage site of the FV molecule resulting in an APC resistance phenotype which is characterized by a poor anticoagulant response of plasma to exogenously added APC [6]. Interestingly, an acquired phenotype mimicking APC resistance is a frequent finding in women using OCs and is believed to be a pathogenic factor of hormone-associated VT [7]–[10].

Inherited APC resistance is associated with increased rates of thrombin formation as demonstrated by increased plasma levels of the prothrombin fragment 1+2 (F1+2) and thrombin-antithrombin complexes (TAT) [11]–[13]. F1+2 is a direct marker of thrombin formation, since it is released from prothrombin during thrombin generation. TAT levels indicate increased rates of thrombin inactivation by the endogenous inhibitor antithrombin (AT). One might speculate that acquired APC resistance similar to the inherited form up-regulates thrombin formation in vivo. There are, however, no data available proving such a hypothesis. If so, quantification of the amount of hormone-associated thrombin formation would be an interesting biomarker candidate to evaluate an increased thrombotic risk associated with hormone intake.

To study the influence of OC on thrombin formation rates in vivo, we measured plasma levels of free thrombin using a recently developed oligonucleotide-based enzyme capture assay (OECA). With a lower limit of quantification (LOQ) of 0.039±0.019 ng/ml it was successfully used to monitor thrombin formation in patients undergoing total hip arthroplasty in a previous study [14]. F1+2, TAT, and other hemostasis parameters were measured additionally. Moreover, plasma levels of APC were quantified using an APC-OECA to detect hormone-associated endothelial cell dysfunction characterized by a dysbalance between thrombin formation and subsequent APC formation [15].

## Materials and Methods

### Subjects

The study was designed as a prospective observational study. Included subjects were healthy women who had not used hormonal contraceptives before. Blood samples were collected between May 2011 and May 2012. Baseline samples were collected before start of OC use (visit 1). After having started taking ethynilestradiol containing OC as prescribed by the women’s gynecologists, further blood samples were collected in the follicular phase of menstruation cycle 2, 3, and 4 after start of OC intake (visits 2–4). 26 out of 32 included women started OC application. 4 women were excluded because OC use was discontinued. In one of these women reason for discontinuation of OC use was detection of the FV-Leiden mutation. One woman was excluded because blood sampling was performed out of schedule.

### Reagents and materials

Human α-thrombin was purchased from CellSystems (St. Katharinen, Germany. rAPC (Xigris) was obtained from Eli Lilly (Indianapolis, USA). Argatroban (Argatra) was obtained from Mitsubishi Pharma (Düsseldorf, Germany). Aprotinin (Trasylol) was obtained from Bayer (Berlin, Germany). Recombinant hirudin (Refludan) was purchased from Pharmion (Hamburg, Germany). Biotinylated aptamers were synthesized and purified by Microsynth (Balgach, Switzerland). The fluorogenic peptide substrates Pefafluor TH (Thrombin, H-D-CHA-Ala-Arg-AMC) and Pefafluor PCa (APC, Pyr-Pro-Arg-AMC) were purchased from Loxo (Dossenheim, Germany).

### Blood sampling

Blood sampling was performed by venipuncture of an antecubital vein using 21-gauge winged infusion sets (Sarstedt, Nümbrecht, Germany). Blood was drawn into citrate tubes (10.5 mM final concentration, Sarstedt, Nümbrecht, Germany) and for thrombin measurement into citrate tubes containing argatroban (100 µmol/l final concentration). For APC measurement citrate tubes were supplemented with aprotinin and r-hirudin (final concentration of 10 µmol/l and 15 µg/ml, respectively). Prior to centrifugation, citrate tubes and thrombin tubes were stored for a maximum time of 2 h at RT. APC tubes were stored on ice (2 h max.). Plasma samples obtained by centrifugation at 2,600×g for 10 min were stored immediately at <−40°C until assayed.

### Laboratory assay procedures

#### Detection of free thrombin and APC by OECA

The OECAs for thrombin and APC detection were performed in the microtiter plate format using white Maxisorp Fluoronunc microtiter modules (Nunc A/S, Roskilde, Denmark) as previously described [14], [15].

Wells were sealed during incubation times with adhesive polyester film (Platemax, Axygen, Union City CA, USA) and stored in the dark. For washing, wells were generally rinsed three times with 300 µl of phosphate-buffered saline (PBS) washing buffer (PBS, 0.05% Tween 20, pH 7.4) using an automated plate washer (SLT Columbus, Tecan, Germany). Wells were initially coated with 10 µg/ml of bovine serum albumin (BSA)-biotin (100 µl/well) in coating buffer (30 mM Na_2_CO_2_, 200 mM NaHCO_3_, pH 9.0) at 4°C overnight. After washing 100 µl of PBS washing buffer containing 1 mg/ml BSA and 10 µg/ml streptavidin were added to the wells and incubated for 1 h at RT. Wells were blocked using 200 µl/well of blocking buffer (PBS, 20 mg/ml BSA, 0.05% Tween 20, pH 7.4). After incubation for 2 h at RT, the remains were aspirated and aptamers loaded.

For loading of aptamers into the streptavidin-coated wells, either 3′-biotinylated thrombin-aptamers HD1–22 for the thrombin-OECA or 3′-biotinylated APC-aptamers HS02-G52 for the APC-OECA were diluted in Tris-buffered saline (TBS; pH 7.6, 1 mmol/l each CaCl_2_ and MgCl_2_, 0.05% Tween 20, 1 mg/ml BSA) and 100 µl of the solution added to the wells of streptavidin-primed modules and incubated at RT for 1 h. After incubation, the wells were washed with TBS washing buffer (TBS, pH 7.6, 1 mmol/l each CaCl_2_ and MgCl_2_, 0.05% Tween 20) and samples or calibrators added (100 µl). Calibration curves covering a ½-log10 concentration range from 0 to 10 ng/ml thrombin (0–272 pmol/l) or rAPC (0–182 pmol/l) were prepared in the corresponding sample matrices and processed in parallel. For the APC OECA, plasma samples and calibrators were re-calcified before analysis by addition of1 mol/l CaCl_2_, yielding a final concentration of 7.5 mmol/l, to improve the binding of APC to the aptamers [15], [16]. After incubation for 2 h at RT, samples and calibrators were removed from the wells using an eight-channel pipette and fresh tips for each column to prevent carry-over contamination during automated washing. Then, 250 µl of PBS-washing buffer were manually added to the wells and the modules washed using the standard PBS-washing procedure.

Subsequently, 100 µL of a fluorogenic substrate solution (100 µmol/l Pefafluor TH or Pefafluor PCa in TBS, pH 8.5, containing 4 mmol/l CaCl_2_) was added to the wells and baseline fluorescence intensities measured using a plate fluorescence reader (FLx-800, Bio-Tek, Bad Friedrichshall, Germany). Changes in fluorescence over time were taken as the measure of thrombin or APC captured in the wells. Data obtained from the calibrators were interpolated by 4-parameter curve fit and used to calculate thrombin or APC concentrations in the samples. Samples and calibrators were assayed in triplicate.

#### Thrombin generation-based evaluation of APC sensitivity

Plasma thrombin generation was initiated by a final tissue-factor concentration of 5 pmol/l in the presence or absence of 5 nmol/l rAPC and monitored by calibrated automated thrombography (CAT) using standard reagents (Thrombinoscope B.V., Maastricht, The Netherlands) and equipment as described elsewhere [17], [18]. For evaluation of APC sensitivity the endogenous thrombin generation potential (ETP) in the presence of rAPC (ETP_+APC_) was divided by the ETP in the absence of rAPC (ETP_−APC_) calculating the APC sensitivity ratio [ETP_+APC_/ETP_−APC_].

#### Other hemostasis parameters

Plasma levels of F1+2 and TAT were determined using the Enzygnost F1+2 (monoclonal) assay, and the TAT micro assay, respectively (Siemens Healthcare Diagnostics Products, Marburg, Germany). Plasma levels of fibrinogen (Clauss method), activity levels of factors (F) II, V, VII, VIII, IX, X, XI, XIII, AT, PC, antigen levels of von Willebrand factor (VWF), and d-dimers were determined using an automated coagulation analyzer (BCS XP, Siemens Healthcare Diagnostics, Eschborn, Germany) and standard reagents (Multifibren U, Innovin, Actin FSL, Berichrom Factor XIII, Berichrom Antithrombin III, Berichrom Protein C, vWF Ag, INNOVANCE D-Dimer; Siemens Healthcare Diagnostics Products, Marburg, Germany). Plasma levels of free protein S (PS) were measured using the HemosIL Free Protein S assay (Instrumentation Laboratory, Bedford, USA). The PAP ELISA assay (DRG Instruments, Marburg, Germany) was used to determine plasma levels of Plasmin-α2-antiplasmin (PAP)-complexes. Plasma levels of tissue-type plasminogen activator (t-PA) antigen were determined using the TECHNOZYM t-PA Ag ELISA assay (Technoclone, Vienna, Austria). All subjects were tested for the FV-Leiden mutation and the prothrombin G20210A mutation using in house methods as previously described [19], [20].

The study proposal was approved by the Institutional Review Board and Ethics committee of the University Hospital of Bonn. Written informed consent was obtained in compliance with the declaration of Helsinki.

### Statistical analysis

Differences between the parameters at the sampling-time points (comparison of visit 2–4 to visit 1) were analyzed using the Wilcoxon signed-rank test after applying the Friedman test to perform ANOVA. Two-tailed tests were used and only values of p<0.0167 were considered significant after Bonferroni correction. Power was calculated retrospectively with α set at 0.05. Correlations were evaluated by Spearman analysis and values of p<0.05 were considered statistically significant.

## Results

### Study population

A total of twenty-one women, mean age 21 years (range 15–40) completed the study. The per-protocol group consisted of all women who took OC as prescribed and had complete measurements of all parameters at all visits. Only subjects of the per-protocol group were included in the statistical analysis. Estrogen-gestagen combinations of the OC used in the per-protocol population are listed in **Table S1**. All women of the per-protocol group were tested negative for the FV-Leiden mutation and the prothrombin G20210A mutation. No thromboembolic events were observed during the study period.

### APC resistance developed in all women during OC use

**Figure 1:**
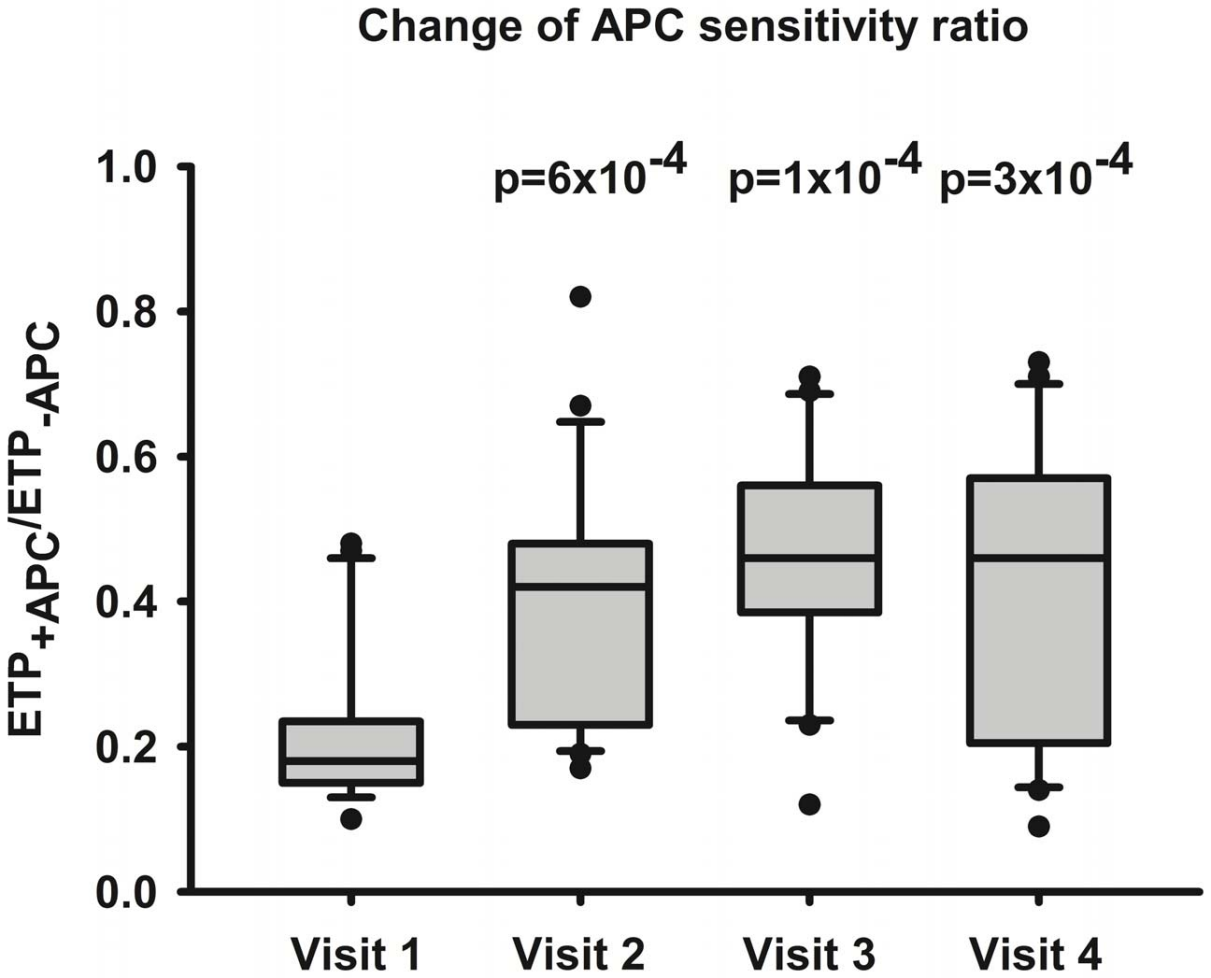
Change of the APC sensitivity ratio during the study period. Box-whisker plots show the ratio [ETP_+APC_/ETP_−APC_] at visits 1–4. The center horizontal solid line is drawn at the median. Top and bottom edges of the boxes are located at the sample 75^th^ and 25^th^ percentiles. Top and bottom of the whiskers are located at the 90^th^ and 10^th^ percentiles. Each outlier is shown. P values indicate the changes of visits 2–4 compared to visit 1.

At baseline a median APC sensitivity ratio [ETP_+APC_/ETP_−APC_] of 0.18 was observed. It increased to 0.42 (p = 0.0006) at visit 2, 0.46 (p = 0.0001) at visit 3, and 0.46 (p = 0.0003) at visit 4, indicating the development of APC resistance during OC use in the study population (**Table S2**, **Fig. 1**). All women showed an increase of the APC sensitivity ratio after the start of OC use, thereof 20 women (95%) at visit 2 and one woman at visit 3. In 17 women (81%) the APC sensitivity ratio remained constantly increased in comparison to baseline during the study period. In three women an increase of the APC sensitivity ratio was observed at visit 2 and 3 followed by a decrease at visit 4. One woman showed a transient increase of the APC sensitivity ratio at visit 2 and 4. During OC use the APC sensitivity ratio was significantly correlated with levels of FVIII (r = 0.5, p = 0.0169 at visit 3; r = 0.493, p = 0.0232 at visit 4) and VWF (r = 0.569, p = 0.0072 at visit 3; r = 0.496, p = 0.0222 at visit 4).

### Free thrombin and APC did not change with OC-induced APC resistance

**Figure 2:**
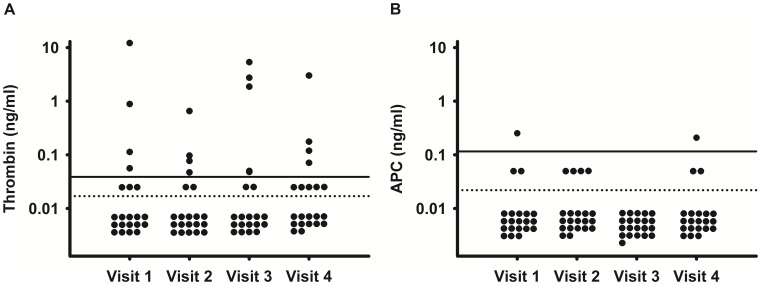
**A.** Thrombin plasma levels, determined by OECA; Limit of quantification (LOQ, solid line), 0.039 ng/ml; Limit of detection (LOD, dotted line), 0.017 ng/ml. **B.** APC plasma levels, determined by OECA; LOQ (solid line), 0.116 ng/ml; LOD (dotted line), 0.022 ng/ml.

At baseline plasma levels of thrombin above the LOQ were detected in four women (19%). Thrombin levels lay between the LOQ and the limit of detection (LOD; 0.017 ng/ml) in three women (14%) and below the LOD in 14 women (67%). A similar pattern was observed at the following visits but the subjects showing thrombin levels above the LOQ changed. No woman showed a constant increase of thrombin levels during the study period or during OC use (**Fig. 2** **A**). Plasma levels of APC above the LOQ were detected in only one woman at baseline and at visit 4 who did not show quantifiable thrombin levels. In all other samples no APC levels >0.116 ng/ml were detected (**Fig. 2** **B**). Overall there were no significant changes of plasma levels of thrombin and APC between visits.

Plasma levels of thrombin were found to be significantly correlated with TAT at all visits during OC use (r = 0.472, p = 0.0310) at visit 2; r = 0.498, p = 0.0213 at visit 3; r = 0.786, 2.4×10^−5^ at visit 4) and with F1+2 at visit 3 (r = 0.476, p = 0.0291). No correlations were found between thrombin and APC levels, d-dimer levels, APC sensitivity ratio, and other parameters. No association was found between plasma levels of thrombin or APC and the type of OC they used. Thrombin and APC plasma levels did not correlate with the subjects’ age.

### Activation markers did not increase during the study period except for PAP

**Figure 3:**
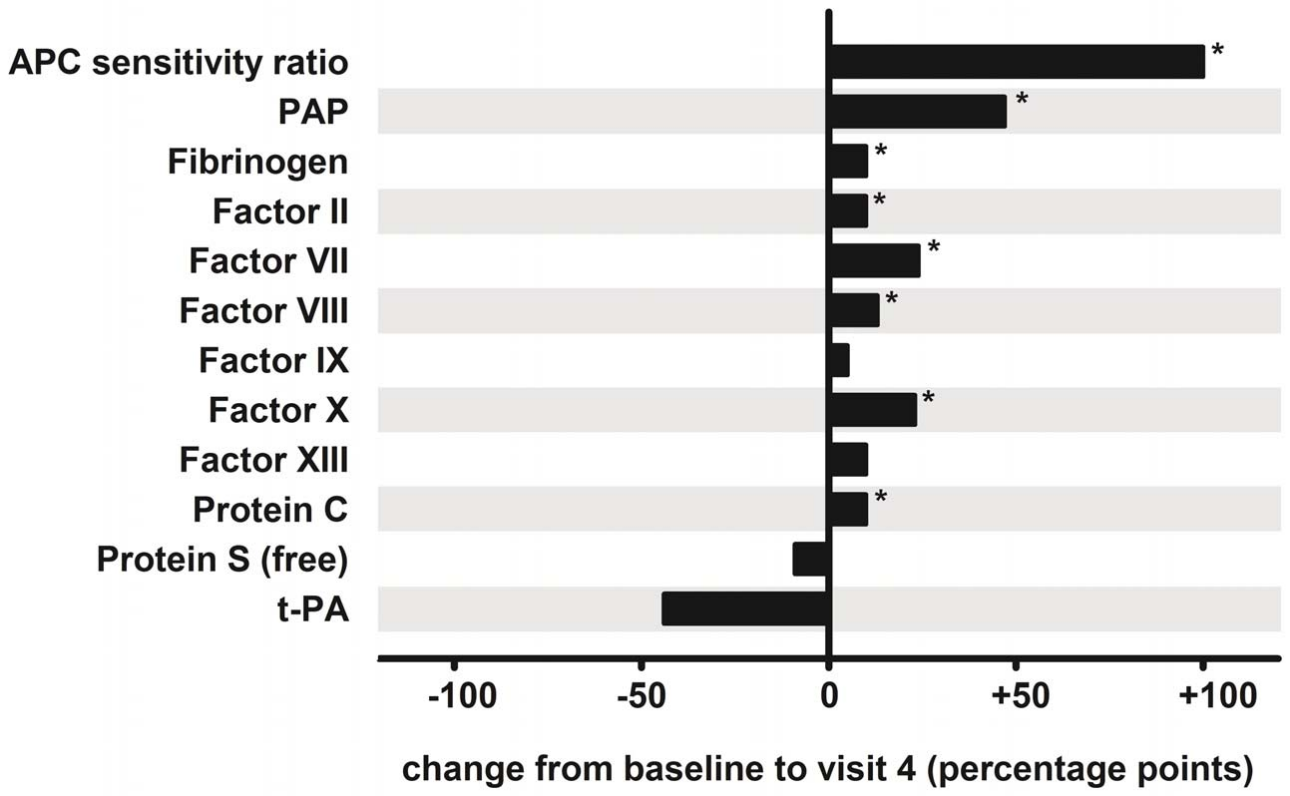
Changes of variables during the study period. Columns show the median. Parameters with significant changes between visit 1 and visit 4 are listed. P values indicate the changes of visits 2–4 compared to visit 1. *indicates highly significant changes of p<0.005.

Significant changes in the analytes during the study period are summarized in **Fig. 3**. Measurements and changes are listed in detail in **Table S2** (APC sensitivity ratio, F1+2, TAT, PAP, d-dimer), **Table S3** (Fibrinogen, FII – FXIII, VWF), and **Table S4** (AT, PC, PS, t-PA).

The indirect markers of thrombin formation F1+2 and TAT did not change significantly during the study period. Median levels lay within their respective reference ranges (<0.34 nmol/l for F1+2, <3.9 ng/ml for TAT) at all visits. Median PAP increased from 439 ng/ml at baseline to 598 ng/ml at visit 2 (p = 0.0034). It further increased to 676 ng/ml at visit 3 (p = 0.0009) and to 678 ng/ml at visit 4 (p = 0.0003), exceeding the upper reference value of 606 ng/ml. After start of OC intake a transient increase of d-dimer levels was observed. Median d-dimer levels increased from 0.29 mg/l at baseline to 0.40 mg/l at visit 2 (p = 0.0105). However, the statistical power calculated for this observed increase was only 0.30. Furthermore, the increase at visits 3–4 was not significant in comparison to baseline and median d-dimer levels were not elevated above the upper reference value of 0.5 mg/l at any visit.

### Procoagulant factors increased under OC

Median fibrinogen was 2.67 g/l at baseline and increased to 3.62 g/l at visit 4 (p = 0.0021), slightly exceeding the upper reference value of 3.55 g/l. Median levels of all other procoagulant factors remained within their respective reference ranges at baseline and subsequent visits. Within these limits the majority of procoagulant factors increased under OC. From visit 1 to visit 4 median FII increased from 113.0 to 132.3% (p = 0.0010), FVII from 101.1 to 132.6% (p = 0.0015), FVIII from 95.7 to 108.3% (p = 0.0018), FIX from 102.6 to 114.9% (p = 0.0147), and FX from 111.6 to 136.7% (p = 0.0005). FXIII also increased significantly from baseline to visit 4 (p = 0.0155). In contrast to the other procoagulant factors, the calculated power for the observed changes in FVIII and FXIII was <0.80. FV, FXI and VWF remained unchanged during the study period.

### PC increased during OC use while free PS and t-PA decreased

An increase of PC after the start of OC use was observed while free PS and t-PA decreased significantly. In comparison to baseline median PC increased from 109.0 to 119.1% at visit 4 (p = 0.0021), free PS decreased from 89.4 to 78.9% (p = 0.0147), and t-PA from 1.02 to <0.60 ng/ml (p = 0.0063), not exceeding the reference ranges of these parameters. With 0.93 only the power calculated for the observed change in PC was >0.80. A transient decrease of AT was observed after start of OC use at visit 2 and visit 3. Compared with baseline values median AT decreased from 109.8 to 98.0% at visit 2 (p = 0.0050) and to 105.9% at visit 3 (p = 0.0121).

## Discussion

This is the first study monitoring plasma levels of enzymatically active thrombin and APC in OC-associated APC resistance. A repeated measures design was used to discriminate between intermittent and constant changes of hemostasis parameters after start of OC use with blood sampling at constant intervals. Blood sampling was performed at the same time points of the menstruation cycle because changes of APC sensitivity during the menstruation cycle have been reported [21]. Preanalytical conditions were strictly controlled ensuring that study results were not influenced by methods of venipuncture, materials used, transport, or storage conditions. The only limitation of the spectrum of OC in our study was the requirement to contain ethynilestradiol. The capacity of various ethynilestradiol containing OC to induce APC resistance has been shown in several previous studies [7]–[10], [22]. Consistent with these reports a thrombin generation-based assay was used for the evaluation of APC sensitivity because of its higher sensitivity in the detection of acquired APC resistance compared with aPTT-based assays [5].

The APC sensitivity ratio increased in all women of our study population during the second or third cycle of OC use and remained increased during the total observation period. This indicates that the development of APC resistance is a constant and stable phenomenon of OC intake. Consistent with previous findings the development of APC resistance was associated with a decrease of free protein S and an simultaneous increase of procoagulant factors including factor VIII and vitamin K-dependent clotting factors [5], [23]. The correlation between APC sensitivity ratio and FVIII underlines the role of FVIII as main determinant of acquired APC resistance [23]. The changes of PAP, fibrinogen, FXIII, PC, and t-PA were also consistent with previous findings in OC users [22], [24]–[27].

Plasma levels of free thrombin, F1+2, and TAT remained unchanged during the whole study period, ruling out that the development of hormone-associated APC resistance up-regulates in-vivo thrombin generation. This is a main difference to inherited APC resistance where an increase of F1+2 and TAT as indicators of an increased thrombin turnover has been reported although plasma levels of free thrombin have not been measured so far [11]–[13].

The plasma concentration of APC-PC-inhibitor (PCI) complexes has also been proposed as marker of coagulation activation in OC users, as increased thrombin activation should cause generation of APC which is inactivated predominantly by complex formation with PCI [28]. APC-PCI complexes were not monitored, but the absence of increased plasma levels of APC gives additional evidence for the absence of increased thrombin formation during OC-induced APC resistance in our study.

Taken together our results demonstrate that the total capacity of thrombin regulation mechanisms remained high enough to prevent a net increase of the thrombin concentration despite OC-associated impairment of the PC pathway. This might explain the rather low thrombotic potential of OC when compared to stronger thrombotic risk factors such as inherited APC resistance, deficiencies of endogenous inhibitors, or major surgery. In a previous study a strong correlation between plasma levels of thrombin and TAT and an increase of thrombin during the course of total hip arthroplasty was observed [14]. In our study thrombin levels were also persistently correlated with TAT during OC use although there was no increase of both parameters.

In conclusion we were able to demonstrate that OC-induced APC resistance is not associated with increased plasma levels of thrombin. Our results reflect the low prothrombotic potential of OC-induced APC resistance and the low incidence of OC-related VT in contrast to high thrombotic risk situations such as major surgery. However, it cannot be ruled out that thrombin formation might increase during OC use in the presence of additional risk factors. As there were no thrombotic events in this study, it cannot be ruled out that increased levels of thrombin could be measured in women suffering from VT during OC use. If this were the case, measurement of endogenous thrombin levels might be helpful to identify OC users at high thrombotic risk. Thus, our data warrant further studies with the occurrence of VT as clinical endpoint and a sufficient number of women to assess the potential value of increased thrombin levels in predicting an increased thrombotic risk.

## Supporting Information

Table S1 **OC used in per-protocol group.**
(DOCX)

Table S2 **Changes of APC sensitivity ratio and activation markers.**
(DOCX)

Table S3 **Changes of procoagulant factors.**
(DOCX)

Table S4 **Changes of anticoagulant and fibrinolytic factors.**
(DOCX)

## References

[1] de Bastos M, Stegeman BH, Rosendaal FR, Van Hylckama Vlieg A, Heimerhorst FM, et al (2014) Combined oral contraceptives: venous thrombosis. Cochrane Database Syst Rev 2014, Mar 3 (3) CD010813 doi: 10.10021/14651858.pub2 10.1002/14651858.CD010813.pub2 PMC10637279 24590565

[2] Manzoli L, De Vito C, Marzuillo C, Boccia A, Villari P (2012) Oral contraceptives and venous thromboembolism: a systematic review and meta-analysis. Drug Saf 35: 191–205. 22283630 10.2165/11598050-000000000-00000

[3] Crawley JT, Zanardelli S, Chion CK, Lane DA (2007) The central role of thrombin in hemostasis. J Thromb Haemost 5 Suppl 195–101. 17635715 10.1111/j.1538-7836.2007.02500.x

[4] Dahlbäck B, Villoutreix BO (2005) The anticoagulant protein C pathway. FEBS Lett 579: 3310–3316. 15943976 10.1016/j.febslet.2005.03.001

[5] Castoldi E, Rosing J (2010) APC resistance: biological basis and acquired influences. J Thromb Haemost 8: 445–453. 20002539 10.1111/j.1538-7836.2009.03711.x

[6] Zöller B, Svensson PJ, He X, Dahlbäck B (1994) Identification of the same factor V gene mutation in 47 out of 50 thrombosis-prone families with inherited resistance to activated protein C. J Clin Invest. 94: 2521–2524. 10.1172/JCI117623 PMC330087 7989612

[7] Rosing J, Tans G, Nicolaes GA, Thomassen MC, van Oerle R, et al (1997) Oral contraceptives and venous thrombosis: different sensitivities to activated protein C in women using second- and third-generation oral contraceptives. Br J Haematol 97: 233–238. 9136971 10.1046/j.1365-2141.1997.192707.x

[8] Rosing J, Middeldorp S, Curvers J, Christella M, Thomassen LG, et al (1999) Low-dose oral contraceptives and acquired resistance to activated protein C: a randomised cross-over study. Lancet 354: 2036–2040. 10636369 10.1016/s0140-6736(99)06092-4

[9] Van Vliet HA, Winkel TA, Noort I, Rosing J, Rosendaal FR (2004) Prothrombotic changes in users of combined oral contraceptives containing drospirenone and cyproterone acetate. J Thromb Haemost 2: 2060–2062. 15550051 10.1111/j.1538-7836.2004.00983.x

[10] Tchaikovski SN, Van Vliet HA, Thomassen MC, Bertina RM, Rosendaal FR, et al (2007) Effect of oral contraceptives on thrombin generation measured via calibrated automated thrombography. Thromb Haemost 98: 1350–1356. 18064335

[11] Zöller B, Holm J, Svensson P, Dahlbäck B (1996) Elevated levels of prothrombin activation fragment 1+2 in plasma from patients with heterozygous Arg506 to Gln mutation in the factor V gene (APC-resistance) and/or inherited protein S deficiency. Thromb Haemost 75: 270–274. 8815575

[12] Simioni P, Scarano L, Gavasso S, Sardella C, Girolami B, et al (1996) Prothrombin fragment 1+2 and thrombin-antithrombin complex levels in patients with inherited APC resistance due to factor V Leiden mutation. Br J Haematol 92: 435–441. 8603014 10.1046/j.1365-2141.1996.d01-1500.x

[13] Gouin-Thibault I, Arkam R, Nassiri S, de la Tourette A, Conard J, et al (2002) Markers of activated coagulation in patients with factor V Leiden and/or G20210A prothrombin gene mutation. Thromb Res 107: 7–11. 12413582 10.1016/s0049-3848(02)00189-5

[14] Müller J, Becher T, Braunstein J, Berdel P, Gravius S, et al (2011) Profiling of active thrombin in human blood by supramolecular complexes. Angew Chem Int Ed Engl 50: 6075–6078. 21591028 10.1002/anie.201007032

[15] Müller J, Friedrich M, Becher T, Braunstein J, Kupper T, et al (2012) Monitoring of plasma levels of activated protein C using a clinically applicable oligonucleotide-based enzyme capture assay. J Thromb Haemost 10: 390–398. 22236082 10.1111/j.1538-7836.2012.04623.x

[16] Müller J, Isermann B, Dücker C, Salehi M, Meyer M, et al (2009) An exosite-specific ssDNA aptamer inhibits the anticoagulant functions of activated protein C and enhances inhibition by protein C inhibitor. Chem Biol 16: 442–451. 19389630 10.1016/j.chembiol.2009.03.007

[17] Hemker HC, Al Dieri R, De Smedt E, Béquin S (2006) Thrombin generation, a function test of the haemostatic-thrombotic system. Thromb Haemost 96: 553–561. 17080210

[18] Castoldi E, Rosing J (2011) Thrombin generation tests. Thromb Res 127: S21–25. 21262433 10.1016/S0049-3848(11)70007-X

[19] Happich D, Schwaab R, Hanfland P, Hoernschemeyer D (1999) Allelic discrimination of factor V Leiden using a 5′ nuclease assay. Thromb Haemost 82: 1294–1296. 10544916

[20] Happich D, Madlener K, Schwaab R, Hanfland P, Pötzsch B (2000) Application of the TaqMan-PCR for genotyping of the prothrombin G20210A mutation and of the thermolabile methylenetetrahydrafolate reductase mutation. Thromb Haemost 84: 144–145. 10928490

[21] van Vliet HA, Rodrigues SP, Snieders MN, van der Meer FJ, Frolich M, et al (2008) Sensitivity to activated protein C during the menstruation cycle in women with and without factor V_Leiden_ . Thromb Res 121: 757–761. 17936879 10.1016/j.thromres.2007.08.010

[22] Tans G, Curvers J, Middeldorp S, Thomassen MC, Meijers JC, et al (2000) A randomized cross-over study on the effect of levonorgestrel- and desogestrel-containing oral contraceptives of the anticoagulant pathways. Thromb Haemost 84: 15–21. 10928463

[23] de Visser MC, van Hylckama Vlieg A, Tans G, Rosing J, Dahm AE, et al (2005) Determinants of the APTT- and ETP-based APC sensitivity tests. J Thromb Haemost 3: 1488–1494. 15978106 10.1111/j.1538-7836.2005.01430.x

[24] Quehenberger P, Kapiotis S, Pärtan C, Schneider B, Wenzel R, et al (1993) Studies on oral contraceptive-induced changes in blood coagulation and fibrinolysis and the estrogen effect on endothelial cells. Ann Hematol 67: 33–36. 8392873 10.1007/BF01709663

[25] Middeldorp S, Meijers JC, van den Ende AE, van Enk A, Bouma BN, et al (2000) Effects on coagulation of levonorgestrel- and desogestrel-containing low dose oral contraceptives: a cross-over study. Thromb Haemost 84: 4–8. 10928461

[26] Meijers JC, Middeldorp S, Tekelenburg W, van den Ende AE, Tans G, et al (2000) Increased fibrinolytic activity during use of oral contraceptives is counteracted by an enhanced factor XI-independent down regulation of fibrinolysis: a randomized cross-over study of two low-dose oral contraceptives. Thromb Haemost 84: 9–14. 10928462

[27] Oral Contraceptive and Hemostasis Study Group (2003) The effects of seven monophasic oral contraceptive regimens on hemostatic variables: conclusions from a large randomized multicenter study. Contraception 67: 173–185. 12618251 10.1016/s0010-7824(02)00476-6

[28] Bremme K, Hamad RR, Berg E, Strandberg K, Stenflo J (2011) The APC-PCI concentration as an early marker of activation of blood coagulation. A study of women on combined oral contraceptives. Thromb Res 130: 636–639. 22154243 10.1016/j.thromres.2011.11.006

