# Supplementary material for: Impact of Hormone-Associated Resistance to Activated Protein C on the Thrombotic Potential of Oral Contraceptives: A Prospective Observational Study
Source: PLoS One. 2014 Aug 14;9(8):e105007. doi: 10.1371/journal.pone.0105007 (PMC4133351; doi:10.1371/journal.pone.0105007)
Supplement: Table S1 — OC used in per-protocol group. (DOCX) [file pone.0105007.s001.docx]

**Table S1 OC used in per-protocol group**

| **Major ingredients** | | **Users, n** |
| --- | --- | --- |
| 20 µg Ethynilestradiol | 100 µg Levonorgestrel | 6 |
| 30 µg Ethynilestradiol | 150 µg Levonorgestrel | 2 |
| 20 µg Ethynilestradiol | 150 µg Desogestrel | 4 |
| 30 µg Ethynilestradiol | 2000 µg Dienogest | 7 |
| 35 µg Ethynilestradiol | 2000 µg Cyproterone acetate | 2 |
