# Supplementary material for: Impact of Hormone-Associated Resistance to Activated Protein C on the Thrombotic Potential of Oral Contraceptives: A Prospective Observational Study
Source: PLoS One. 2014 Aug 14;9(8):e105007. doi: 10.1371/journal.pone.0105007 (PMC4133351; doi:10.1371/journal.pone.0105007)
Supplement: Table S2 — Changes of APC sensitivity ratio and activation markers. (DOCX) [file pone.0105007.s002.docx]

**Table S2 Changes of APC sensitivity ratio and activation markers**

| **Parameter** | **Visit** | **median** | **P25** | **P75** | **p** | **power** |
| --- | --- | --- | --- | --- | --- | --- |
| **ETP_+APC_/ETP_-APC_** | 1 | 0.18 | 0.15 | 0.22 |  |  |
|  | 2 | 0.42 | 0.24 | 0.46 | 0.0006 | 0.95 |
|  | 3 | 0.46 | 0.40 | 0.55 | 0.0001 | 0.96 |
|  | 4 | 0.46 | 0.21 | 0.57 | 0.0003 | 0.95 |
| **F1+2 (nmol/l)** | 1 | 0.12 | 0.10 | 0.16 |  |  |
|  | 2 | 0.13 | 0.10 | 0.18 | ns |  |
|  | 3 | 0.13 | 0.11 | 0.16 | ns |  |
|  | 4 | 0.15 | 0.12 | 0.17 | ns |  |
| **TAT (ng/ml)** | 1 | 2.41 | <2.00 | 2.78 |  |  |
|  | 2 | 2.35 | <2.00 | 2.82 | ns |  |
|  | 3 | 2.56 | <2.00 | 3.04 | ns |  |
|  | 4 | 2.60 | 2.03 | 4.38 | ns |  |
| **PAP (ng/ml)** | 1 | 439 | 271 | 546 |  |  |
|  | 2 | 598 | 447 | 843 | 0.0034 | 0.89 |
|  | 3 | 676 | 632 | 916 | 0.0009 | 0.95 |
|  | 4 | 678 | 571 | 925 | 0.0003 | 0.95 |
| **D-Dimer (mg/l)** | 1 | 0.29 | 0.26 | 0.41 |  |  |
|  | 2 | 0.40 | 0.32 | 0.57 | 0.0105 | 0.30 |
|  | 3 | 0.35 | 0.31 | 0.78 | ns |  |
|  | 4 | 0.37 | 0.31 | 0.61 | ns |  |

P25, 25^th^ percentile; P75, 75^th^ percentile; ns, not significant.
