# Supplementary material for: Impact of Hormone-Associated Resistance to Activated Protein C on the Thrombotic Potential of Oral Contraceptives: A Prospective Observational Study
Source: PLoS One. 2014 Aug 14;9(8):e105007. doi: 10.1371/journal.pone.0105007 (PMC4133351; doi:10.1371/journal.pone.0105007)
Supplement: Table S3 — Changes of procoagulant factors. (DOCX) [file pone.0105007.s003.docx]

**Table S3 Changes of procoagulant factors**

| **Parameter** | **Visit** | **median** | **P25** | **P75** | **p** | **power** |
| --- | --- | --- | --- | --- | --- | --- |
| **Fibrinogen (g/l)** | 1 | 2.67 | 2.39 | 3.36 |  |  |
|  | 2 | 3.25 | 2.43 | 3.79 | 0.0042 | 0.76 |
|  | 3 | 3.28 | 2.58 | 3.61 | ns |  |
|  | 4 | 3.62 | 2.94 | 4.09 | 0.0021 | 0.80 |
| **FII (%)** | 1 | 113.0 | 106.1 | 125.0 |  |  |
|  | 2 | 126.3 | 117.4 | 135.9 | 0.0002 | 0.95 |
|  | 3 | 126.3 | 118.5 | 141.9 | 0.0008 | 0.95 |
|  | 4 | 132.3 | 120.3 | 143.5 | 0.0010 | 0.93 |
| **FV (%)** | 1 | 119.2 | 102.8 | 124.8 |  |  |
|  | 2 | 98.5 | 92.1 | 114.3 | ns |  |
|  | 3 | 109.5 | 96.4 | 123.1 | ns |  |
|  | 4 | 117.1 | 99.1 | 130.3 | ns |  |
| **FVII (%)** | 1 | 101.1 | 86.7 | 121.4 |  |  |
|  | 2 | 121.8 | 94.0 | 132.5 | 0.0071 | 0.85 |
|  | 3 | 128.4 | 111.5 | 139.4 | 0.0053 | 0.82 |
|  | 4 | 132.6 | 113.9 | 144.0 | 0.0015 | 0.94 |
| **FVIII (%)** | 1 | 95.7 | 82.9 | 104.9 |  |  |
|  | 2 | 104.3 | 85.6 | 119.7 | 0.0019 | 0.86 |
|  | 3 | 97.2 | 83.0 | 117.3 | ns |  |
|  | 4 | 108.3 | 95.5 | 122.9 | 0.0018 | 0.75 |
| **FIX (%)** | 1 | 102.6 | 91.9 | 107.1 |  |  |
|  | 2 | 109.0 | 98.4 | 110.9 | ns |  |
|  | 3 | 109.4 | 100.5 | 117.1 | 0.0065 | 0.85 |
|  | 4 | 114.9 | 97.8 | 121.8 | 0.0147 | 0.84 |
| **FX (%)** | 1 | 111.6 | 100.2 | 127.4 |  |  |
|  | 2 | 129.2 | 112.5 | 141.6 | 0.0070 | 0.95 |
|  | 3 | 135.5 | 122.2 | 149.5 | 0.0003 | 0.95 |
|  | 4 | 136.7 | 122.8 | 149.0 | 0.0005 | 0.95 |
| **FXI (%)** | 1 | 98.0 | 92.7 | 105.4 |  |  |
|  | 2 | 100.5 | 97.3 | 106.7 | ns |  |
|  | 3 | 101.9 | 94.1 | 112.2 | ns |  |
|  | 4 | 107.3 | 99.5 | 115.6 | ns |  |
| **FXIII (%)** | 1 | 108.4 | 98.7 | 114.4 |  |  |
|  | 2 | 112.6 | 100.5 | 135.2 | ns |  |
|  | 3 | 121.0 | 100.7 | 133.1 | ns |  |
|  | 4 | 107.4 | 100.7 | 132.7 | 0.0155 | 0.72 |
| **VWF (%)** | 1 | 95.0 | 69.3 | 120.8 |  |  |
|  | 2 | 106.8 | 57.5 | 129.5 | ns |  |
|  | 3 | 96.2 | 61.7 | 152.7 | ns |  |
|  | 4 | 108.0 | 68.3 | 131.4 | ns |  |

P25, 25^th^ percentile; P75, 75^th^ percentile; ns, not significant.
