# Supplementary material for: Impact of Hormone-Associated Resistance to Activated Protein C on the Thrombotic Potential of Oral Contraceptives: A Prospective Observational Study
Source: PLoS One. 2014 Aug 14;9(8):e105007. doi: 10.1371/journal.pone.0105007 (PMC4133351; doi:10.1371/journal.pone.0105007)
Supplement: Table S4 — Changes of anticoagulant and fibrinolytic factors. (DOCX) [file pone.0105007.s004.docx]

**Table S4 Changes of anticoagulant and fibrinolytic factors**

| **Parameter** | **Visit** | **median** | **P25** | **P75** | **p** | **power** |
| --- | --- | --- | --- | --- | --- | --- |
| **AT (%)** | 1 | 109.8 | 103.3 | 116.4 |  |  |
|  | 2 | 98.0 | 92.7 | 105.4 | 0.0050 | 0.86 |
|  | 3 | 105.9 | 96.3 | 112.5 | 0.0121 | 0.77 |
|  | 4 | 109.4 | 103.1 | 114.5 | ns |  |
| **PC (%)** | 1 | 109.0 | 98.9 | 121.4 |  |  |
|  | 2 | 112.4 | 100.3 | 125.2 | ns |  |
|  | 3 | 114.9 | 106.2 | 126.2 | ns |  |
|  | 4 | 119.1 | 104.5 | 133.3 | 0.0021 | 0.93 |
| **Free PS (%)** | 1 | 89.4 | 84.0 | 99.4 |  |  |
|  | 2 | 86.0 | 77.3 | 97.9 | ns |  |
|  | 3 | 83.7 | 70.7 | 92.9 | ns |  |
|  | 4 | 78.9 | 68.8 | 93.4 | 0.0147 | 0.68 |
| **t-PA (ng/ml)** | 1 | 1.02 | <0.60 | 1.42 |  |  |
|  | 2 | 0.74 | <0.60 | 1.09 | ns |  |
|  | 3 | <0.60 | <0.60 | 0.97 | ns |  |
|  | 4 | <0.60 | <0.60 | 0.79 | 0.0063 | 0.64 |

P25, 25^th^ percentile; P75, 75^th^ percentile; ns, not significant.
